# Cilia Stimulatory and Antibacterial Activities of T2R Bitter Taste Receptor Agonist Diphenhydramine: Insights into Repurposing Bitter Drugs for Nasal Infections

**DOI:** 10.3390/ph15040452

**Published:** 2022-04-06

**Authors:** Li Eon Kuek, Derek B. McMahon, Ray Z. Ma, Zoey A. Miller, Jennifer F. Jolivert, Nithin D. Adappa, James N. Palmer, Robert J. Lee

**Affiliations:** 1 Department of Otorhinolaryngology, University of Pennsylvania Perelman School of Medicine, Philadelphia, PA 19104, USA; li_eon@icloud.com (L.E.K.); zoeym@pennmedicine.upenn.edu (Z.A.M.); jennifer.jolivert@pennmedicine.upenn.edu (J.F.J.); nithin.adappa@pennmedicine.upenn.edu (N.D.A.); james.palmer@pennmedicine.upenn.edu (J.N.P.); 2 Harriton High School, Bryn Mawr, PA 19010, USA; s015446@students.lmsd.org; 3 Department of Physiology, University of Pennsylvania Perelman School of Medicine, Philadelphia, PA 19104, USA

**Keywords:** *Staphylococcus aureus*, *Pseudomonas aeruginosa*, ciliary beat frequency, calcium, nitric oxide, G protein-coupled receptors, cystic fibrosis, chronic rhinosinusitis

## Abstract

T2R bitter taste receptors in airway motile cilia increase ciliary beat frequency (CBF) and nitric oxide (NO) production. Polymorphisms in some T2Rs are linked to disease outcomes in chronic rhinosinusitis (CRS) and cystic fibrosis (CF). We examined the expression of cilia T2Rs during the differentiation of human nasal epithelial cells grown at air–liquid interface (ALI). The T2R expression increased with differentiation but did not vary between CF and non-CF cultures. Treatment with Pseudomonas aeruginosa flagellin decreased the expression of diphenhydramine-responsive T2R14 and 40, among others. Diphenhydramine increased both NO production, measured by fluorescent dye DAF-FM, and CBF, measured via high-speed imaging. Increases in CBF were disrupted after flagellin treatment. Diphenhydramine impaired the growth of lab and clinical strains of *P. aeruginosa*, a major pathogen in CF and CF-related CRS. Diphenhydramine impaired biofilm formation of *P. aeruginosa*, measured via crystal violet staining, as well as the surface attachment of *P. aeruginosa* to CF airway epithelial cells, measured using colony-forming unit counting. Because the T2R agonist diphenhydramine increases NO production and CBF while also decreasing bacterial growth and biofilm production, diphenhydramine-derived compounds may have potential clinical usefulness in CF-related CRS as a topical therapy. However, utilizing T2R agonists as therapeutics within the context of *P. aeruginosa* infection may require co-treatment with anti-inflammatories to enhance T2R expression.

## 1. Introduction

Every inhalation is an opportunity for pathogens to enter the airways. Thus, airway epithelia have multiple protective countermeasures [1,2]. Mucus secreted by goblet cells entraps pathogens while multi-ciliated cells move pathogens out of the airway via mucociliary clearance (MCC) [3]. MCC is compromised in cystic fibrosis (CF) [4] and other “muco-obstructive” lung diseases [5]. CF is characterized by dehydrated airway surface liquid (ASL) and thickened mucus, preventing pathogen removal and leading to respiratory infections (6). Mucociliary clearance is also disrupted in chronic rhinosinusitis (CRS) due to thickened mucus and/or cilia dysfunction [6]. Enhancing MCC may aid host defenses in diseases such as CF or CRS [7].

Because antibiotic-resistant organisms are common in patients with CF and CRS [8,9,10,11,12,13,14,15,16], this may complement or reduce therapy with conventional antibiotics. CRS, a complex syndrome of upper airway inflammation and/or infection, affects >35 million Americans [17,18,19,20,21,22,23] and leads to ~20% of adult antibiotic prescriptions in the US [19,20,21,22]. Many CF patients also develop a type of CRS termed CF-related CRS (CF-CRS) [24]. CF-CRS impairs patient quality of life, and CF nasal infections can seed potentially fatal lower respiratory infections [25]. Topical therapies to boost MCC and/or kill bacteria are of particular interest in the nose, where tissue is accessible to high volume nasal rinse (e.g., neti pot) strategies [26].

Cilia are the first contact points for airway pathogens and are equipped with receptors to recognize these pathogens [3]. Several members of the G protein-coupled bitter taste receptors (taste family 2 receptors or T2Rs) localize to airway cilia [27,28] and can detect bacterial products, including acyl-homoserine lactones [29] and quinolones [28] secreted by opportunistic airway pathogen *Pseudomonas aeruginosa*. While first identified on the tongue, T2Rs are expressed all over the body where they serve diverse chemosensory roles involved in immunity and metabolism [30,31]. In the airways, cilia T2Rs increase ciliary beat frequency (CBF) via calcium-activated nitric oxide (NO) production and protein kinase G (PKG) phosphorylation of ciliary proteins [32] and the NO produced also has antibacterial effects against *P. aeruginosa* [33].

Polymorphisms rendering the cilia-localized T2R38 isoform non-functional are correlated with increased nasal bacterial load, susceptibility to CRS, and worse sinus surgery outcomes in non-CF patients (reviewed in [3]). These same polymorphisms are correlated with more severe nasal symptoms in CF-CRS patients [34] and may have relevance to lower airway infections in CF [35]. CF-CRS patients are often infected with *P. aeruginosa* [24,25]. Because *P. aeruginosa* is particularly sensitive to NO-dependent killing compared with other nasal bacteria such as Staphylococcus aureus [33], targeting T2Rs may perhaps be most beneficial in CF-CRS where the bacteria are most sensitive to the innate immune responses activated. We hypothesize that activating T2Rs may be a strategy to enhance MCC or bactericidal NO-generation in CF-CRS.

We sought to examine whether diphenhydramine (DPD), a T2R14 agonist [36,37] as well as H1 antihistamine, targets T2Rs in nasal cilia to induce NO production and CBF increases. T2R14 is one of the more highly expressed T2R in nasal and lung epithelial cells [28]. Importantly, DPD is water soluble up to the mM range, and thus DPD or derived compounds (e.g., non-absorbable lectin or dextran conjugates) could be delivered in localized high doses to nasal tissue, which may be useful in a transient stimulation situation as would occur with a high-volume rinse. We report that the T2R expression increases with mucociliary differentiation, but T2R expression is downregulated by Toll-like receptor 5 (TLR5) agonist flagellin and IL-4 treatment. T2R14 is among the higher expressed T2R isoforms. T2R14 agonist DPD increases NO production and ciliary beating; however, this response is greatly reduced after TLR5 priming. Additionally, DPD reduces *P. aeruginosa* growth and biofilm formation, suggesting potent T2R-independent benefits of DPD or derived compounds. This paper supports the idea that screening-known bitter clinical drugs may result in identification T2R agonists that have both cilia/NO stimulatory effects and antibacterial activities.

## 2. Results

### 2.1. T2R Expression Increases with Nasal Mucociliary Differentiation but Is Decreased by P. aeruginosa Flagellin

**Figure 1:**
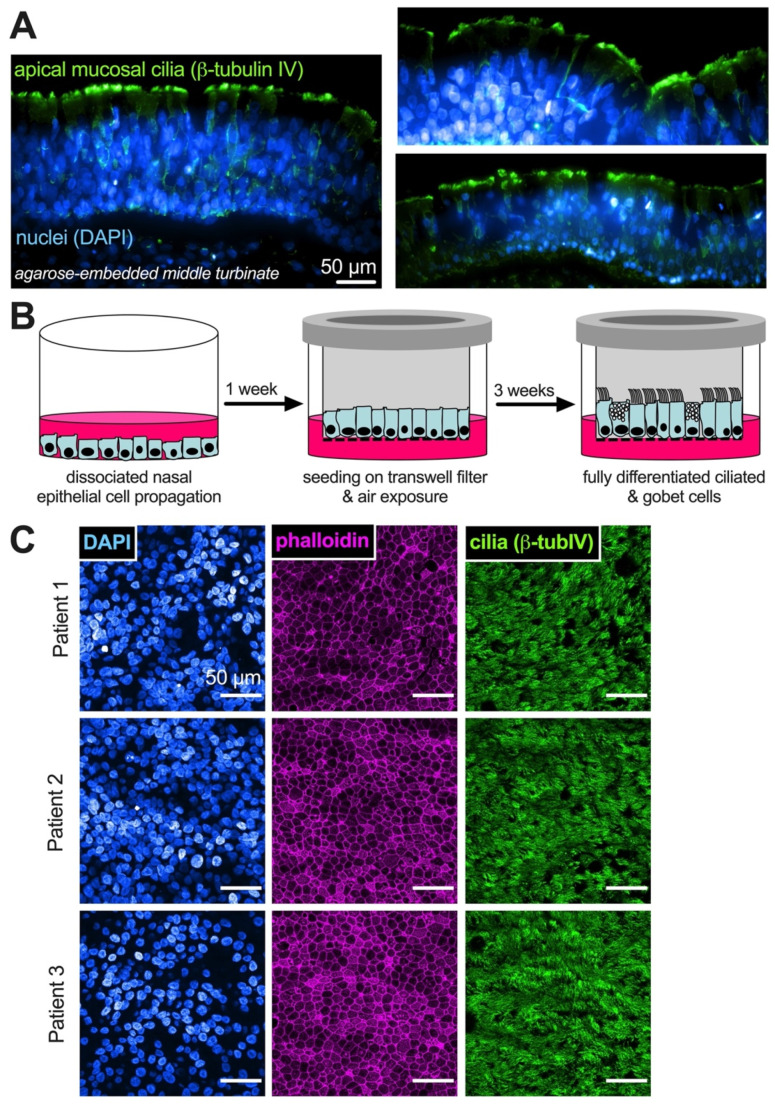
Generation of air–liquid interface (ALI) cultures from residual surgical material. (**A**) Nasal turbinate mucosal tissue was removed from the bone, embedded in agarose, sliced and fixed. Immunofluorescence staining for β-tubulin IV (green) shows the highly ciliated mucosal surface. Nuclei are shown in blue. Representative tissue slices from three patients are shown. Spinning disk confocal, 10 × 0.4 NA objective. Scale bar is 50 µm. (**B**) non-fixed or embedded tissue is enzymatically dissociated and basal epithelial cells are propagated in submersion culture. Cells are seeded onto permeable Transwell filters at high density, and the next day, the medium on the apical surface is removed. The resulting air exposure on the apical side leads to the differentiation of cells into ciliated and goblet cells reminiscent of the in vivo epithelium; (**C**) representative immunofluorescence of ALI cultures derived from three different patients after three weeks of differentiation, showing an abundance of ciliated cells (β-tubulin IV; green). AlexaFluor 647-labeled phalloidin staining of actin (magenta) shows tight cobblestone epithelial pattern and apical microvilli. Nuclear DAPI stain shown in blue. Images taken on laser-scanning confocal, 60×. Images are maximum z-projections. Scale bar is 50 µm.

We first tested whether the expression of some T2Rs increases with mucociliary differentiation in vitro. Primary nasal epithelial cells were isolated from residual middle turbinate from sinus surgery (Figure 1A) cultured in propagation medium that expands the basal cell population and then differentiated at the air–liquid interface (Figure 1B). Upon air exposure, cultured primary human nasal epithelial basal cells differentiate to ciliated cells (Figure 1B) which contain several bitter taste receptor isoforms (T2Rs) that act as sentinels in the innate immune system [28].

**Figure 2:**
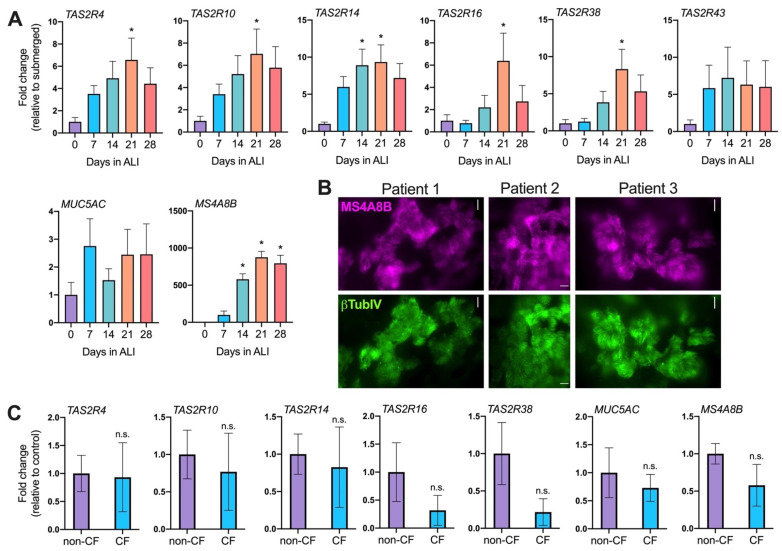
T2R expression in primary nasal air–liquid interface (ALI) cultures. (**A**) T2R expression increases during differentiation of nasal ALIs. Ciliary T2Rs, 4, 10, 14, 16 and 38 mRNA expression increased with at least 3 weeks of differentiation, as revealed by qPCR. Note that T2R receptors are encoded by corresponding *TAS2R* genes. Data points represent the average of 5 patients; significance was analyzed by one-way ANOVA using Dunnett’s post-test (* *p* < 0.05). (**B**) Immunofluorescence of MS4A8B (Mouse monoclonal antibody 3E6, Kerafast, Boston, MA) and β-tubulin IV (Rabbit monoclonal EPR16775, Abcam) showing cilia co-localization. Representative images shown from 3 ALIs from 3 individual patients. (**C**) Cystic fibrosis does not alter T2R mRNA expression. Cilia-localized T2Rs 4, 10, 14, 16 and 38 did not significantly alter the expression when comparing nasal ALIs derived from either non-CF or CF patients. Cilia marker MS4A8B and MUC5AC expression demonstrates that cultures were differentiated. Bar graphs represent data obtained from 5 non-CF and 3 CF patients. Data pairs were analyzed by Student’s t-test showing no significant differences.

Differentiation of non-CF nasal air–liquid interface cultures (ALIs) was determined by the increase in cilia marker MS4A8B [3,38] which increased to a maximal after 3–4 weeks of exposure to air (Figure 2A,B) as well as the formation of high transepithelial electrical resistance (Supplementary Figure S1). Transcripts for cilia-localized T2R’s, including T2R14, increased over the same time course in non-CF ALIs (Figure 2A). No significant difference was observed between the T2R transcript levels in non-CF vs. F508del/F508del homozygous CF ALIs after differentiation (Figure 2C) at day 21. While there was a trends toward decreased *TAS2R38* expression, this was not significant. This fits with the published gene array data suggesting T2R expression is similar between non-CF and CF nasal cells in vivo (Supplementary Figure S2).

**Figure 3:**
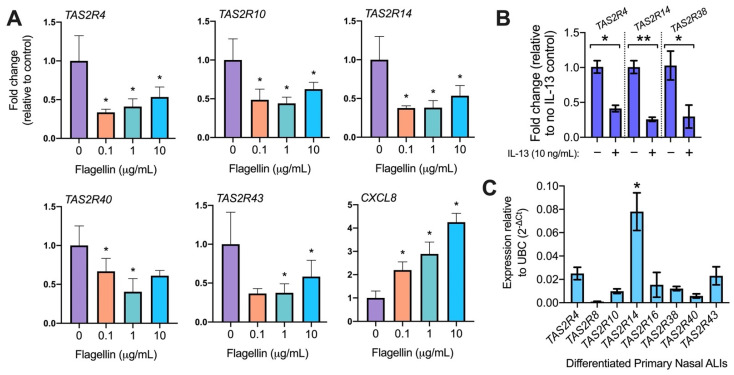
Changes in T2R expression with inflammatory mediators. (**A**) Flagellin decreases T2R expression in nasal ALIs. Human nasal epithelial cells were isolated and grown in ALI cultures and then exposed to air to induce differentiation for 4 weeks prior to treatment. Cultures were treated with various concentrations of flagellin (0.1, 1 or 10 µg/mL) for 24 h prior to RNA analysis via qPCR. Bar graphs represent combined data from 4 patients. Data were analyzed by one-way ANOVA, Dunnett’s post-test; * *p* < 0.05. (**B**) IL-13 treatment decreases T2R expression in nasal ALIs. Human nasal epithelial cells were isolated and grown in ALI cultures then exposed to air to induce differentiation for 3 weeks prior to treatment. Data were analyzed by one-way ANOVA, Bonferroni post-test; * *p* < 0.05 and ** *p* < 0.01. Bar graph represents combined data from 4 patients. (**C**) Expression of T2Rs in 3-week post-air primary nasal ALIs normalized to housekeeping gene UBC using 2^−ΔCt^ method where ΔCt = Ct_GAPDH_ − Ct_target gene_. Data shown are the mean ± SEM from ALIs grown from 15 different individual patients. TAS2R14 expression was significantly higher than other TAS2R genes examined by one-way ANOVA with Bonferroni post-test; * *p* < 0.05.

This initially suggested that T2Rs may be therapeutic targets in CF nasal cells as proposed in non-CF nasal cells [3]. However, we also surprisingly found that *P. aeruginosa* flagellin, a TLR5 agonist [39], reduced the expression of T2R14 and other T2R isoforms (Figure 3A). Differentiated nasal ALIs were treated with media containing 0.1, 1, or 10 µg/mL of flagellin overnight. Exposure to flagellin downregulated T2R4, 10, 14, 40, and 43 transcripts while IL-8 (*CXCL8*) expression increased, as expected (Figure 3A). The T2R expression decrease in response to flagellin was eliminated when a recombinant soluble TLR5 ectodomain protein was included to bind and block the flagellin (Supplementary Figure S3). Flagellin had no effect on T2R expression in Beas-2B cells (Supplementary Figure S4A), suggesting that this response is specific for primary differentiated cells. We also observed that TLR4 agonist lipopolysaccharide (LPS) had no effect on T2R expression in primary nasal cells despite being able to increase the IL-6 and IL-8 transcript (Supplementary Figure S4B). This suggests that the decreased T2R response may be specific for TLR5. However, we found that primary ALIs’ differentiated in vitro expressed significantly more (~10×) TLR5 compared with TLR4 (Supplementary Figure S4C). An alternative explanation is that LPS is elevating IL-6 or IL-8 in these cultures through another target (e.g., an intracellular target [40]) and TLR4 expression is too low to regulate T2R expression.

We also observed that five days of treatment with a Th2 cytokine implicated in nasal polyp inflammation, IL-13 [41,42], markedly down-regulated cilia-localized T2R4, 14, and 38 expression in well-differentiated (day 21) nasal ALIs (Figure 3B). This fits with the marked downregulation of cilia and the up-regulation of Muc5AC positive goblet cells observed after IL-13 treatment in vitro [43]. Together, these data suggest that T2R expression can be modified by a variety of inflammatory factors within the context of sinonasal inflammation. In bronchial epithelial cells, previously shown to express T2Rs [27], we also observed a similar increase in T2R expression with mucociliary differentiation (Supplementary Figure S5A) and likewise a decrease in T2R expression with flagellin treatment (Supplementary Figure S5B). Thus, studies of T2Rs in the nasal cavity may have utility in understanding lung epithelial physiology as well.

### 2.2. Diphenhydramine Activates NO Production and Increases CBF in Nasal Epithelial Cells

**Figure 4:**
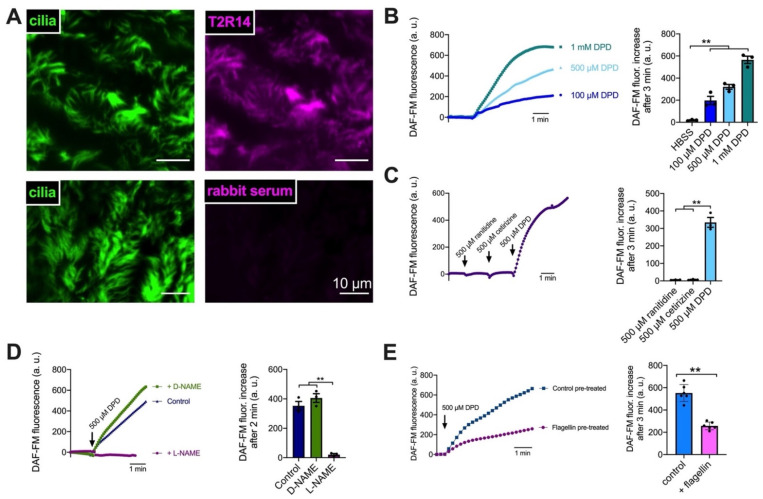
Diphenhydramine induces nitric oxide (NO) production in nasal ALIs. (**A**) T2R14 localizes to cilia in nasal ALIs. Representative immunofluorescence image of nasal cilia (β-tubulin IV; green) and T2R14 (magenta) from ALI cultures from two individual donors. No staining was observed with rabbit serum plus secondary control (bottom). (**B**) NO production, as revealed by DAF-FM fluorescence, increased in a dose-dependent manner with diphenhydramine (DPD). (**C**) Anti-histamines ranitidine and cetirizine did not initiate NO production, while equimolar diphenhydramine did. (**D**) NOS inhibitor L-NAME (10 µM) blocked NO production while negative control D-NAME (10 µM) did not. (**E**) Flagellin pre-treatment resulted in less NO production in response to DPD. Representative traces from ≥3 experiments using ALIs from different non-CF patients are shown. Bar graphs are mean ± SEM analyzed via one-way ANOVA (**B**–**D**) or Student’s *t* test (**E**); ** *p* < 0.01.

Compared with housekeeping gene UBC, we found that T2R14 is highest expressed among the known nasal epithelial cilia-localized T2R isoforms (T2Rs 4, 14, 16, and 38) (Figure 3C). T2R14 is expressed in both bronchial [27] and nasal [44] cilia (Figure 4A). T2R14 is a receptor activated by a broad range of pharmaceutical drugs with established safety data [37,45,46] as well as plant compounds [37,47]. The wealth of T2R14 agonists means that this receptor might be more easily exploited to activate innate immune responses, particularly if we can find agonists that also have antibacterial effects. We hypothesized that finding an agonist with high solubility that could be delivered at high doses might enhance any antibacterial effects.

As described above, T2Rs in cilia activate NO production [44]. We tested whether NO production is activated by diphenhydramine (DPD), a commonly used H1 antihistamine that is also a T2R14 and 40 agonist [37]. As expected from a T2R agonist, DPD (100 µM–1 mM) activated NO production in nasal ALIs as visualized via NO-sensitive dye DAF-FM fluorescence (Figure 4B). Equimolar H1 or H2 antagonists cetirizine or ranitidine, respectively, which are not known to activate T2R14, did not activate NO production (Figure 4C). Thus, this is likely an effect of T2R stimulation rather than H1 antagonism. NO synthase (NOS) inhibitor L-NAME (10 µM, 1 h pretreatment) inhibited DPD-induced NO production, while the negative control D-NAME did not (Figure 4D). Fitting with flagellin lowering T2R14 transcript (Figure 2A), when primary nasal ALIs were treated with flagellin (0.1 µg/mL) overnight, the DAF-FM fluorescence responses were reduced by ~50% (Figure 4E).

**Figure 5:**
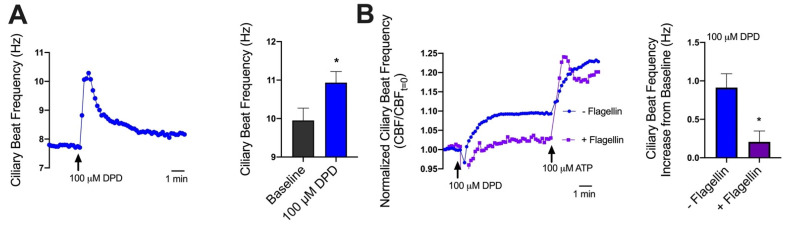
Flagellin represses DPD enhancement of ciliary beat frequency. (**A**) T2R14 agonist diphenhydramine (100 µM) increases CBF by up to 1 Hz. (**B**) Fully differentiated (day 21) nasal ALI cultures were treated with media containing 0.1 µg/mL flagellin for 72 h to ensure reduction in both RNA and protein expression. Flagellin-treated cultures did not reveal a 1 Hz increase in CBF when treated with diphenhydramine. Traces are from 1 patient representative of results from ≥3 patients. Bar graphs are combined data from a ≥3 ALIs from ≥3 individual patients. * *p* < 0.05.

T2R NO production increases CBF [44]. DPD (100 µM) increased CBF by ~1 Hz (~10%; Figure 5A), comparable with T2R-induced CBF increases in other studies [47,48]. Overnight stimulation with flagellin (0.1 µg/mL, which reduced T2R14 expression Figure 2A) caused a loss of the DPD-induced CBF increase (Figure 5B) but not the purinergic receptor-driven ATP-activated increase (Figure 3B). These results suggest that DPD CBF increases are due to T2R activation. Moreover, they suggest that while T2Rs can be activated for beneficial NO and CBF responses in CF cells, other inflammatory factors released by *P. aeruginosa* may limit T2R responses by down-regulating T2R expression. Notably, we found that neither cetirizine nor azelastine at equimolar concentration (100 µM) elevated CBF (Supplementary Figure S6A–D). The CBF increase with DPD stimulation was reduced by 6-methoxyflavanone (Supplementary Figure S6E), a T2R14 and T2R39 antagonist [49] previously shown to reduce T2R14/DPD Ca^2+^ responses [50].

### 2.3. Diphenhydramine Inhibits P. aeruginosa Growth, Biofilm Production, and Attachment to CFBE Cells

**Figure 6:**
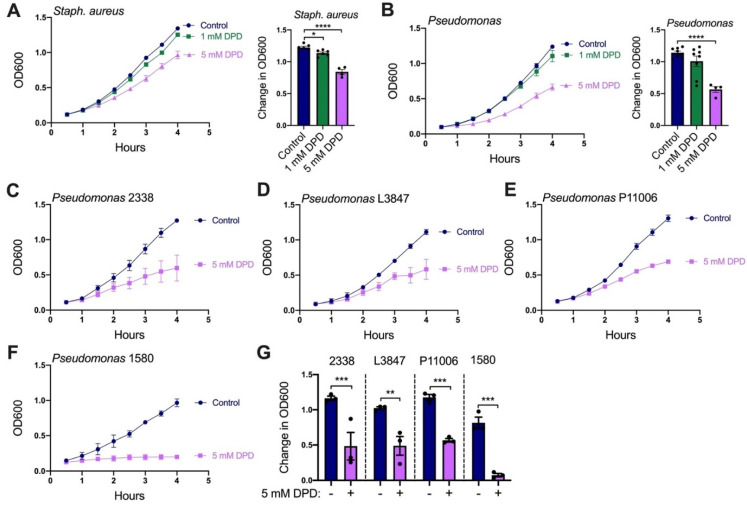
Diphenhydramine represses the planktonic growth of *S. aureus* and *P. aeruginosa*. (**A**) *S. aureus* strain M2 growth (measured by OD_600_) was reduced by 1–5 mM diphenhydramine. (**B**) DPD (5 mM) significantly decreased the growth of *Pseudomonas* lab strain PAO-1. (**C**–**F**) The growth of clinical strains of *Pseudomonas* 2338 (**C**), L3847 (**D**), P11006 (**E**) and 1580 (**F**) were all impaired by 5 mM DPD. (**G**) Bar graph summarizing results from (**C**–**F**). Traces are representative of ≥3 experiments with mean ± SEM in bar graphs analyzed via one-way ANOVA using Sidak’s post-test (* *p* < 0.05, ** *p* < 0.01, *** *p* < 0.001, **** *p* < 0.0001).

DPD inhibited planktonic growth of both *Staphylococcus aureus* (methicillin-resistant lab strain M2; Figure 6A) and *P. aeruginosa* (PAO1; Figure 6B). We further explored *P. aeruginosa* because of its relevance to CF and CF-CRS as well as the larger effect of DPD on *P. aeruginosa* compared with *S. aureus*. We found that DPD also reduced the planktonic growth of clinical strains of *P. aeruginosa* isolated from CF-CRS patients (Figure 6C–G).

**Figure 7:**
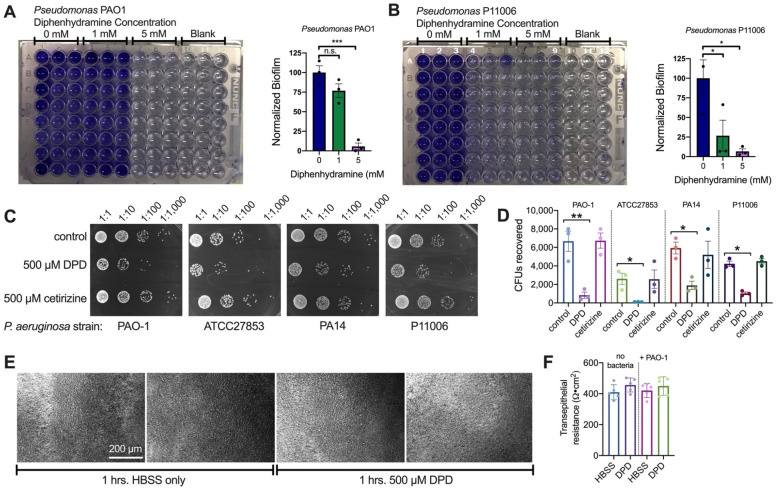
Diphenhydramine (DPD) inhibits *P. aeruginosa* biofilm production and attachment to CFBE cells. (**A**,**B**) Representative images of crystal violet staining showing diphenhydramine-reduced biofilm mass at 5 mM for lab strain PAO1 (**A**) and 1 mM for clinical strain P11006 (**B**). Other clinical strains used in Figure 4 did not form biofilms on 96-well plates and so could not be used in this assay. Representative images of the 96-well plate are shown. Data from three experiments are combined in bar graphs analyzed by one-way ANOVA using Dunnett’s post-test (* *p* < 0.05, *** *p* < 0.001). (**C**) Representative images of CFU counts following CFBE attachment assays as described in the text. DPD (500 µM) reduced the amount of bacteria recovered from CFBE cells while equimolar cetirizine did not. Control cells stimulated with media only. Lab strains PAO-1, ATCC27853 and PA-14 and clinical strain P11006 were used. (**D**) Quantification of results from independent experiments as in C. Significance by one-way ANOVA with Bonferroni post-test comparing all values to respective control; * *p* < 0.5, ** *p* < 0.01. (**E**) Representative phase contrast images of CFBE ALIs incubated in HBSS ± 500 µM DPD (1 h; 37 °C). No signs of epithelial break down were observed. Representative of 6 ALIs per condition from independent experiments. (**F**) Transepithelial resistance was measured ±DPD stimulation as in E. No significant difference was observed by one-way ANOVA.

DPD (1–5 mM) also inhibited biofilm production of *P. aeruginosa* PAO1 and clinical CRS *P. aeruginosa* isolate P11006, as visualized through crystal violet staining (Figure 7A,B). We tested whether this affected *P. aeruginosa* binding to CFBE41o- (CFBE) cells using a standardized bacterial attachment assay [51]. CFBE cells are F508del/F508del SV-40-immortalized bronchial epithelial cells. CFBE cells expressed both T2R14 and eNOS and exhibit Ca^2+^ and NO responses with DPD stimulation (Supplementary Figure S7), suggesting that they can approximate both lower and upper airway epithelial cells. CFBE cells were incubated with several lab strains of *P. aeruginosa* and P11006 ± DPD on the apical side, followed by the washing and recovery of adherent bacteria which were quantified by CFU counting. DPD caused 5–10-fold less bacteria to adhere to the CFBE cells (Figure 7C,D). No toxic effects of DPD were observed in CFBE ALIs upon the visual inspection of the integrity of culture monolayers (Figure 7E) or by changes in transepithelial resistance (Figure 7F).

## 3. Discussion

Targeting cilia T2Rs may activate CBF and NO-dependent bacterial killing in CF-related CRS or non-CF CRS patients (20). While many CF patients are greatly helped by CFTR ion channel corrector/potentiator therapies, those with a premature stop codon (e.g., G542X) and other mutations cannot benefit from these therapies [52]. We hypothesized that targeting T2Rs may be a particularly useful strategy for eradicating *P. aeruginosa* infections in these CF and/or CF-CRS patients for two reasons. First, the endogenous agonists known to activate airway cilia T2Rs (acyl-homoserine lactones and quinolones [3]) are produced by *P. aeruginosa*. Second, *P. aeruginosa* are more sensitive to NO-dependent killing than *Staphylococcus aureus* [33]. This suggests that the cilia T2Rs may have evolved to detect the bacteria most susceptible to this defensive pathway. Further work is needed to clarify the interactions of specific bacterial species with cilia T2Rs.

The T2R expression peaked at 21 days and then appeared to decrease somewhat. This may reflect the experimental variation inherent in the use of human samples and a lack of power to detect significant differences at day 28. Alternatively, it may reflect a biological phenomenon (e.g., the “aging” of the cultures or a transient peak of T2R expression during cilia differentiation). Either case requires future studies using cultures derived from larger patient populations. We found that T2R expression is similar between CF and non-CF patients in the absence of inflammatory stimuli. However, inflammation in CF may impair the signaling of some T2Rs. We saw decreased T2R14 expression and NO/cilia responses to T2R14 agonist diphenhydramine after *Pseudomonas* flagellin pretreatment. The effects of flagellin were not dose-dependent in the range of 0.1–10 µg/mL; the values all reduced T2R expression in most cases, suggesting that the effects had already plateaued, despite a continued dose-dependent increase in IL-8 expression at the same flagellin concentrations. This may reveal clues to the mechanism of flagellin reduction in T2R14 for future studies. The reduction in T2R expression with flagellin was surprising; we initially expected an increase in T2R expression since T2Rs are tied to innate defense. NFκB signaling downstream of TLR5 may shift the main defensive pathway towards an increased production of antimicrobial peptides such as defensins or enhanced NO production via inducible (i)NOS. Nonetheless, the patient gene expression array data (Supplementary Figure S2) suggested no major differences between nasal T2R expression between patients with mild or severe CF vs. non-CF patients. However, greater differences might be observed if CF patients were stratified by *P. aeruginosa* nasal infection status. Further patient profiling is needed.

T2R14, the highest expressed T2R investigated here, is activated by plant flavones and *P. aeruginosa* quinolones [3], competence stimulating peptides from *Streptococcus mutans* [53], and many pharmaceutical drugs [45,50]. We hypothesize that T2R14 is an ideal isoform to target because it is highly expressed, as found in other tissues [54]. Additionally, T2R14 does not have the same high prevalence of polymorphisms detrimental to the receptor function observed with T2R38 [3]. These so-called “non-taster” polymorphisms would render many patients non-responsive to drugs targeting T2R38. T2R14 is also an attractive T2R to target in the nose because of its broad range of agonist drugs from both synthetic pharmaceutical drugs [37,45,46] and plant products [55,56,57,58].

DPD, a T2R14 agonist in addition to first-generation H1 antihistamine, induces T2R innate immune responses, increasing CBF and NO production. DPD also disrupts *P. aeruginosa* growth, biofilm production and the cell attachment of both lab and clinical strains. DPD was shown to have antibacterial effects against *S. aureus* [59] and may potentiate the effects of levofloxacin against *S. aureus* or *P. aeruginosa* [60]. We showed effects of DPD alone against *P. aeruginosa* at concentrations that activate T2R receptors. We previously showed the antibacterial activity of other T2R14 agonist plant flavones [61]. We are not suggesting the use of intranasal DPD. DPD is not likely an optimal T2R agonist to use clinically due to the drowsiness and other off-target effects (e.g., anticholinergic activity) induced by first generation H1 antihistamines [62]. However, DPD has a much higher water solubility than other ciliostimulatory T2R agonists such as flavones, and DPD is already inexpensively produced. We hypothesize that a DPD-related compound, for example, a modified non-absorbable form of DPD (e.g., a dextran or lectin conjugate), could maintain both bitterness and anti-bacterial effects and might be useful and logistically feasible to include in a nasal rinse to treat Gram-negative infections. Thus, further work is needed. Because histamine does not measurably activate nasal epithelial ciliary beating in nasal ALIs [63], the antihistamine effects of such a compound would not be expected to have any significant detrimental effect on ciliary beating.

While topically used antihistamine azelastine tastes bitter to some patients [64], it does not increase ciliary beating. Both azelastine and levocabastine may cause irreversible ciliostasis at therapeutic concentrations and cause reversible decreases of cilia beating at diluted concentrations [65]. Other studies support the ciliostatic effects of azelastine [66,67,68] while two other studies suggest that azelastine itself may not be ciliostatic but that rather the benzalkonium preservative used in clinical formulations is ciliostatic [69,70]. Regardless, azelastine does not activate NO production from the human nasal cultures, described above, and thus would not be expected to have the same immune-boosting effects as a DPD-derived compound. While one study suggested a mixture of azelastine and fluticasone mimicked airway dilation observed with T2R agonist chloroquine in mice [71], T2R-like effects on human nasal epithelial cells were not reported. Differences between T2R responses in mouse and human nasal cells (reviewed in [3]) may contribute to a lack of effects of azelastine on human nasal T2Rs.

While other studies have shown an enhancement of antibiotic effects with diphenhydramine against several bacteria [59,60,72,73]. To our knowledge, this is the first demonstration of antibiofilm activity. While the mechanism for the effects of DPD on bacteria must be investigated in future work, these observations suggest a potential dual action in the airway by activating the host’s innate immunity via T2R NO production and decreasing bacterial growth and biofilm formation via an unknown mechanism. Our data also suggest that finding other novel T2R agonists with antibacterial effects is likely. We previously observed that plant flavones as well as T2R14 agonists have anti-bacterial effects against *P. aeruginosa* [61]. Thus, it may be that the evolution of T2Rs as a bacterial detection system has resulted in their detection of many compounds somewhat similar to bioactive bacterial compounds that affect microbial physiology.

Notably, every patient tested in this study responded to DPD. We observed similar global responses with T2R14 plant agonists in our ALI model system [47]. While polymorphisms in *TAS2R13*, *TAS2R19*, *TAS2R38* and *TAS2R49* have been associated with CRS in genome-wide association studies [74], the *TAS2R14* gene has not to our knowledge been associated with CRS. More specific genetic analyses of *TAS2R14* with CRS or other airway diseases may be warranted. While *TAS2R14* does not appear to have known polymorphisms that alter responses to DPD, it has been well demonstrated in vitro that T2R bitter taste receptor isoforms can dimerize with other T2R isoforms [75,76] and possibly other GPCRs [77]. With the majority of GPCRs, the functional consequences of homo- or hetero-dimerization are still unclear [78,79] but may influence ligand–receptor interactions [79]. Polymorphisms in some T2R isoforms may exist that affect the function of hetero-oligomeric binding partners including T2R14. While T2R4 localizes to the tip of nasal motile cilia exclusive from T2R14 [27,80], T2R14 closely co-localizes with T2R38 and T2R16 [80]. We did observe that CBF responses to T2R14 agonist flavones are not different in cultures genotyped as *TAS2R38* PAV/PAV or AVI/AVI homozygotes [47]. However, most *TAS2R* gene polymorphisms are found well below the Mendelian frequency of the common *TAS2R38* PAV and AVI polymorphisms. Thus, future work from a larger genotyped patient population is needed to explore the potential influence of these other *TAS2R* polymorphisms on nasal cilia T2R function.

## 4. Materials and Methods

### 4.1. Reagents

DAF-FM and Fluo-4-AM were from Invitrogen (Grand Island, NY, USA). Anti-beta-tubulin IV (mouse monoclonal ab11315) anti-T2R38 (rabbit polyclonal ab130503) were from Abcam (Cambridge, MA, USA). Anti-T2R14 (PA5-39710; rabbit polyclonal) was from ThermoFisher Scientific (Waltham, MA, USA) and anti-eNOS antibody (NB-300–605; rabbit polyclonal) was from Novus (Littleton, CO, USA). L- and D-NG-nitroarginine methyl ester (L-NAME and D-NAME) were from Cayman (Ann Arbor, MI, USA). All other reagents were obtained from Sigma-Aldrich (St. Louis, MO, USA). Working solutions of diphenhydramine (DPD), cetirizine and azelastine were made immediately prior to use in Hank’s balanced salt solution (HBSS) with no DMSO.

### 4.2. Primary Nasal Epithelial Cell Culture

Primary nasal epithelial cell culture was as described [28] in accordance the U.S. Department of Health and Human Services Title 45 CFR 46.116, the Declaration of Helsinki and University of Pennsylvania guidelines for residual clinical material (Institutional review board #800614). Written informed consent was obtained from patients ≥ 18 years of age undergoing surgery for CRS or trans-nasal approaches to the skull base for tumor removal. Nasal epithelial cells were obtained through enzymatic dissociation tissue in minimal essential media (MEM; ThermoFisher, Waltham, MA, USA) containing 1.4 mg/mL pronase (MilliporeSigma, St. Louis, MO, USA) and 0.1 mg/mL DNase (MilliporeSigma) for 1 h at 37 °C, followed by 2 washes with MEM plus 10% fetal bovine serum. Cells were then incubated on tissue culture plastic in PneumaCult-Ex Plus (Cat.# 05040, Stemcell Technologies, Vancouver, BC, Canada) containing 100 U/mL penicillin and 100 μg/mL streptomycin (Gibco, Waltham, MA, USA) at 37 °C, 5% CO_2_ for 2 h to allow for the adherence and removal of non-epithelial cells (e.g., fibroblasts, macrophages and lymphocytes). Nasal epithelial cells were transferred to a 10 cm tissue culture dish containing PneumaCult-Ex Plus overnight. The following day, culture medium was aspirated and replaced with fresh PneumaCult-Ex Plus. Nasal primary cells were passaged every 4–5 days or when 80% confluent. Cells were seeded for air–liquid interface culture (0.33 cm^2^ Transwell filters, 0.4 µm pore size, transparent) as described [28]). Upon aspiration of apical media and apical air exposure (37 °C, 5% CO_2_ incubator air), cells were fed every other day with Pneumacult ALI media on the basolateral side only. For stimulation with flagellin or LPS, we used *P. aeruginosa* flagellin (FLA-PA, InvivoGen, San Diego, CA, USA) and LPS (L8643, Millipore Sigma).

### 4.3. CFBE and Beas-2B Cell Culture

Parental CFBE41o- (not overexpressing Wt or ΔF508 CFTR) were previously provided by D. Greunert (UCSF) and grown in minimal essential media (MEM) plus Earle’s salts containing (ThermoFisher) 1× cell culture pen/strep (Gibco), 1 mM L-glutamine (Gibco), 10% FetalPlex serum substitute (Gemini Biosciences, West Sacramento, CA, USA). Cells were cultured for 5 days on Transwell filters (1 cm^2^ surface area; 0.4 µm pore size, transparent, Greiner BioOne, Kremsmünster, Austria) before apical aspiration of media and exposure to air. Cells were fed basolaterally for an additional 7 days before being used to establish a high transepithelial electrical resistance (TEER) and tight epithelial barrier. ALIs were fed on the basolateral side with the same media used for cell propagation. Beas-2B cells were cultured in submersion in Ham’s F12K media (ThermoFisher) plus 10% FBS and 1% Pen/Strep.

### 4.4. Live Cell Imaging of Intracellular Calcium (Ca^2+^), Nitric Oxide (NO) Production and Ciliary Beat Frequency (CBF)

Imaging of NO and CBF was as described [28]. For NO, ALI cultures were loaded with DAF-FM by incubation in 10 μM DAF-FM diacetate (ThermoFisher) on the apical side in HBSS plus 5 μM carboxy-PTIO (to scavenge baseline NO; Cayman Chemical) for 90 min. For submerged CFBEs on 8-well chambered coverglass (CellVis, Sunnyvale, CA, USA), loading was the same but for 45 min instead of 90 min. After washing, imaging was performed using an IX-83 microscope (10x 0.4 NA PlanApo objective, Olympus Life Sciences, Tokyo, Japan) with LED illumination (Excelitas Technologies LED120Boost), 16-bit Orca Flash 4.0 sCMOS camera (Hamamatsu, Japan), standard FITC filter set (470/40 nm excitation, 495 lp dichroic, and 525/40 nm emission; 49002-ET, Chroma Technologies) and MetaFluor (Molecular Devices, Sunnyvale, CA, USA). For cilia beating, cultures were maintained at ~28 °C in DPBS (+1.8 mM calcium) on the apical side and HEPES-buffered HBSS supplemented with 1× MEM amino acids (Gibco) on the basolateral side. Sisson-Ammons Video Analysis software was used to measure whole-field CBF. Diphenhydramine and all other reagents used were from Millipore Sigma unless otherwise specified.

### 4.5. Quantitative Reverse Transcription PCR (qPCR)

ALIs were lysed in TRIzol (ThermoFisher). RNA was isolated using Direct-zol (Zymo Research). RNA was transcribed to cDNA via High-Capacity cDNA Reverse Transcription Kit (ThermoFisher); cDNA was then quantified utilizing Taqman Q-PCR probes (QuantStudio 5 Real-Time PCR System, ThermoFisher). Data were then analyzed using Microsoft Excel and plotted in GraphPad PRISM.

### 4.6. Bacterial Assays

In all bacterial assays, growth and biofilm were carried out as described [61]. *Pseudomonas aeruginosa* lab strain PAO-1 was from American Type Culture Collection (HER-1018; ATCC BAA-47). Clinical isolates from *P. aeruginosa* from CF-related CRS were obtained from Dr. Noam Cohen and Dr. Laura Chandler from the Philadelphia VA Medical Center Department of Otorhinolaryngolgoy and clinical microbiology lab, respectively. Planktonic *P. aeruginosa* or methicillin-resistant *S. aureus* M2 [61] strains were cultured in LB (ThermoFisher) at 37 °C with shaking. For planktonic growth, an overnight log-phase culture was diluted to OD 0.1 in 10 mL total volume per sample. Cultures were grown at 37 °C with shaking (180 RPM); 1 mL of solution was removed at each time point and assayed for OD at 600 nm in a spectrophotometer.

*P. aeruginosa* biofilms were grown at 37 °C (without shaking) in 25% LB (low nutrient media to promote biofilm formation) for 5 days in standard 96 well microtiter plates. OD 0.1 cultures were diluted 1:100 in 25% LB and 100 µL was added to each well of a 96-well plate. Plates were sealed with parafilm to prevent dehydration. After incubation, microtiter plates were washed three times with distilled water, followed by staining with 1% crystal violet in water for approximately 30 min. After second washing, biofilm mass and crystal violet were solubilized by 30% acetic acid for 30 min with gentle occasional shaking, and read on a plate reader (Spark 10 M, Tecan) at 590 nm.

Bacterial attachment assays were carried out similarly to [51]. CBFE ALI cultures (12-well plate size, 1 cm^2^ surface area) were washed on the basolateral side with PBS and transferred into antibiotic-free media 48 h before use in these assays. The apical side was washed three times with PBS before use. *P. aeruginosa* were grown overnight, diluted to OD 0.1 in sterile serum-free DMEM with shaking for 1 h at 37 °C. CFBE ALIs were inoculated on the apical side with 3 × 10^6^ CFU in 0.5 mL in media ± diphenhydramine ± ceterizine. Co-cultures were incubated for 1 h at 37 °C, followed by washing the apical side 3x with sterile media and a further 4 h incubation with 500 µL sterile media on the apical side. Cultures were then washed again with sterile media on the apical side. Remaining adherent bacteria were removed by washing with sterile PBS + 1% triton X-100. This solution was then serially diluted 3 times and 10 µL was spotted on LB plates for CFU counts.

### 4.7. Immunofluorescence Microscopy

ALI cultures were fixed in 4% paraformaldehyde for 20 min at room temperature. Blocking and permeabilization was in DPBS + 5% normal donkey serum, 1% BSA, 0.2% saponin and 0.3% Triton X-100 for 45 min at 4 °C. Cultures were incubated in primary antibody at 1:100 in blocking buffer (no triton) at 4 °C overnight. Cultures were then incubated with AlexaFluor-labeled donkey anti-mouse or anti-rabbit (1:1000) at 4 °C for 1 h. ALIs were cut out and mounted with Fluoroshield with DAPI mounting media (Abcam, Cambridge, MA). Images were taken on an Olympus IX-83 microscope using x60 objective (1.4 NA oil; MetaMorph software). T2R14 (PA5-39710) was from ThermoFisher.

Submerged CFBEs were cultured on collagen-coated glass (MatTek, Ashland, MA, USA) and fixed in 3.2% paraformaldehyde for 10 min followed by blocking/permeabilization in DPBS + 5% normal donkey serum, 1% BSA, 0.2% saponin and 0.1% Triton X-100 for 30 min at 4 °C. Primary antibodies used were for GLUT-1 (mouse monoclonal SPM498; MS-10637-P0; LabVision, San Francisco, CA, USA), endothelial (e) NOS (NOS3; SC-654 rabbit polyclonal, SantaCruz Biotechnology, Dallas TX, USA), inducible (i) NOS (NOS2; NB300-605S5) and neuronal (n) NOS (NOS1; ab5586).

### 4.8. Data Analysis and Statistics 

Analyses were performed in Excel or GraphPad Prism; *p* < 0.05 was considered statistically significant. One-way analysis of variance (ANOVA) was performed with Dunnett’s post-test (comparisons to control group), Tukey–Kramer post-test (for comparisons of all samples) or Bonferroni post-test (preselected pair-wise comparisons). All bar graphs are mean ± SEM; * indicates *p* < 0.05 and ** indicates *p* < 0.01. Images were analyzed in ImageJ (W. Rasband, NIH, Bethesda, MD, USA). All data from human primary ALIs were obtained from separate ALIs grown from cells obtained from ≥3 different individual patients as indicated in the figure legends. Control and experimental conditions were tested using ALIs derived from the same patients (patient-matched).

## 5. Conclusions

In summary, we characterized the expression changes of *TAS2R* genes in primary nasal cells with mucociliary differentiation and inflammatory mediators. We showed that, across cells from multiple patients, T2R14 is the highest expressed among the known cilia T2Rs. We observed that T2R14 agonist DPD increased both CBF and NO production in nasal ALIs, consistently with T2R activation. However, the responses to DPD were inhibited by the priming of cells with *P. aeruginosa* flagellin, likely due to TLR5 signaling. The full benefits of T2R activation may require co-stimulation with anti-inflammatories to reduce TLR signaling within the context of CF inflammation and/or *P. aeruginosa* infection to enhance T2R expression. However, DPD inhibits *P. aeruginosa* growth, biofilm production and attachment to lung epithelial cells through mechanisms that remain to be determined. This dual action of DPD or derived compounds may have therapeutic applications in CRS and/or CF-CRS nasal infections.

## Data Availability

Data are contained within the article and supplementary material.

## Supplementary Materials

The following supporting information can be downloaded at: https://www.mdpi.com/article/10.3390/ph15040452/s1, Figure S1: Transepithelial electrical resistance (TEER) of primary air–liquid interface (ALI) nasal cultures; Figure S2: TAS2R gene expression in non-CF vs. CF nasal epithelium; Figure S3: Inhibition of Flagellin effects with hTLR5-Fc.; Figure S4: Flagellin-induced changes in TAS2R expression are specific to primary cells and specific to flagellin; Figure S5: TAS2R gene expression in bronchial cells; Figure S6: Diphenhydramine (DPD) increases ciliary beat frequency while cetirizine (CTZ) and azelastine (AZL) do not.; Figure S7: Diphenhydramine (DPD) responses in CFBE41o- (CFBE) cells [28,43,47,50,81,82,83,84,85].

## References

[1] Hariri B.M., Cohen N.A. (2016). New insights into upper airway innate immunity. Am. J. Rhinol. Allergy.

[2] Parker D., Prince A. (2011). Innate immunity in the respiratory epithelium. Am. J. Respir. Cell Mol. Biol..

[3] Kuek L.E., Lee R.J. (2020). First contact: The role of respiratory cilia in host-pathogen interactions in the airways. Am. J. Physiol. Lung Cell Mol. Physiol..

[4] Saint-Criq V., Gray M.A. (2017). Role of CFTR in epithelial physiology. Cell. Mol. Life Sci..

[5] Boucher R.C. (2019). Muco-Obstructive Lung Diseases. N. Engl. J. Med..

[6] Gudis D., Zhao K.Q., Cohen N.A. (2012). Acquired cilia dysfunction in chronic rhinosinusitis. Am. J. Rhinol. Allergy.

[7] Antunes M.B., Gudis D.A., Cohen N.A. (2009). Epithelium, cilia, and mucus: Their importance in chronic rhinosinusitis. Immunol. Allergy Clin. N. Am..

[8] Bhattacharyya N., Kepnes L.J. (2008). Assessment of trends in antimicrobial resistance in chronic rhinosinusitis. Ann. Otol. Rhinol. Laryngol..

[9] Genoway K.A., Philpott C.M., Javer A.R. (2011). Pathogen yield and antimicrobial resistance patterns of chronic rhinosinusitis patients presenting to a tertiary rhinology centre. J. Otolaryngol. Head Neck Surg..

[10] Kingdom T.T., Swain R.E. (2004). The microbiology and antimicrobial resistance patterns in chronic rhinosinusitis. Am. J. Otolaryngol..

[11] Casey G. (2012). Antibiotics and the rise of superbugs. Kai Tiaki Nurs. N. Z..

[12] Hawser S. (2012). Surveillance programmes and antibiotic resistance: Worldwide and regional monitoring of antibiotic resistance trends. Antibiotic Resistance.

[13] Foucault C., Brouqui P. (2007). How to fight antimicrobial resistance. FEMS Immunol. Med. Microbiol..

[14] Alanis A.J. (2005). Resistance to antibiotics: Are we in the post-antibiotic era?. Arch. Med. Res..

[15] Domin M.A. (1998). Highly virulent pathogens—A post antibiotic era?. Br. J. Theatre Nurs..

[16] Falagas M.E., Bliziotis I.A. (2007). Pandrug-resistant Gram-negative bacteria: The dawn of the post-antibiotic era?. Int. J. Antimicrob. Agents.

[17] Bhattacharyya N. (2011). Incremental health care utilization and expenditures for chronic rhinosinusitis in the United States. Ann. Otol. Rhinol. Laryngol..

[18] Bhattacharyya N., Grebner J., Martinson N.G. (2012). Recurrent acute rhinosinusitis: Epidemiology and health care cost burden. Otolaryngol. Head Neck Surg..

[19] Blackwell D.L., Collins J.G., Coles R. (2002). Summary health statistics for U.S. adults: National Health Interview Survey, 1997. Vital Health Stat..

[20] Ly N., McCaig L.F. (2002). National Hospital Ambulatory Medical Care Survey: 2000 outpatient department summary. Adv. Data Vital Health Stat..

[21] Ray N.F., Baraniuk J.N., Thamer M., Rinehart C.S., Gergen P.J., Kaliner M., Josephs S., Pung Y.H. (1999). Healthcare expenditures for sinusitis in 1996: Contributions of asthma, rhinitis, and other airway disorders. J. Allergy Clin. Immunol..

[22] Cherry D.K., Woodwell D.A. (2002). National Ambulatory Medical Care Survey: 2000 summary. Adv. Data.

[23] Fokkens W.J., Lund V.J., Mullol J., Bachert C., Alobid I., Baroody F., Cohen N., Cervin A., Douglas R., Gevaert P. (2012). European Position Paper on Rhinosinusitis and Nasal Polyps 2012. A summary for otorhinolaryngologists. Rhinology.

[24] Chaaban M.R., Kejner A., Rowe S.M., Woodworth B.A. (2013). Cystic fibrosis chronic rhinosinusitis: A comprehensive review. Am. J. Rhinol. Allergy.

[25] Kang S.H., Dalcin P.d.T.R., Piltcher O.B., Migliavacca R.d.O. (2015). Chronic rhinosinusitis and nasal polyposis in cystic fibrosis: Update on diagnosis and treatment. J. Bras. Pneumol..

[26] Zhao K., Craig J.R., Cohen N.A., Adappa N.D., Khalili S., Palmer J.N. (2016). Sinus irrigations before and after surgery-Visualization through computational fluid dynamics simulations. Laryngoscope.

[27] Shah A.S., Ben-Shahar Y., Moninger T.O., Kline J.N., Welsh M.J. (2009). Motile cilia of human airway epithelia are chemosensory. Science.

[28] McMahon D.B., Kuek L.E., Johnson M.E., Johnson P.O., Horn R.L.J., Carey R.M., Adappa N.D., Palmer J.N., Lee R.J. (2022). The bitter end: T2R bitter receptor agonists elevate nuclear calcium and induce apoptosis in non-ciliated airway epithelial cells. Cell Calcium.

[29] Jaggupilli A., Singh N., Jesus V.C., Duan K., Chelikani P. (2018). Characterization of the Binding Sites for Bacterial Acyl Homoserine Lactones (AHLs) on Human Bitter Taste Receptors (T2Rs). ACS Infect. Dis..

[30] Harmon C.P., Deng D., Breslin P.A.S. (2021). Bitter Taste Receptors (T2Rs) are Sentinels that Coordinate Metabolic and Immunological Defense Responses. Curr. Opin. Physiol..

[31] Jeruzal-Swiatecka J., Fendler W., Pietruszewska W. (2020). Clinical Role of Extraoral Bitter Taste Receptors. Int. J. Mol. Sci..

[32] Salathe M. (2007). Regulation of mammalian ciliary beating. Annu. Rev. Physiol..

[33] Workman A.D., Carey R.M., Kohanski M.A., Kennedy D.W., Palmer J.N., Adappa N.D., Cohen N.A. (2017). Relative susceptibility of airway organisms to antimicrobial effects of nitric oxide. Int. Forum Allergy Rhinol..

[34] Adappa N.D., Workman A.D., Hadjiliadis D., Dorgan D.J., Frame D., Brooks S., Doghramji L., Palmer J.N., Mansfield C., Reed D.R. (2016). T2R38 genotype is correlated with sinonasal quality of life in homozygous DeltaF508 cystic fibrosis patients. Int. Forum Allergy Rhinol..

[35] Castaldo A., Cernera G., Iacotucci P., Cimbalo C., Gelzo M., Comegna M., Di Lullo A.M., Tosco A., Carnovale V., Raia V. (2020). TAS2R38 is a novel modifier gene in patients with cystic fibrosis. Sci. Rep..

[36] Robinett K.S., Koziol-White C.J., Akoluk A., An S.S., Panettieri R.A., Liggett S.B. (2014). Bitter taste receptor function in asthmatic and nonasthmatic human airway smooth muscle cells. Am. J. Respir. Cell Mol. Biol..

[37] Meyerhof W., Batram C., Kuhn C., Brockhoff A., Chudoba E., Bufe B., Appendino G., Behrens M. (2010). The molecular receptive ranges of human TAS2R bitter taste receptors. Chem. Senses.

[38] Eon Kuek L., Leffler M., Mackay G.A., Hulett M.D. (2016). The MS4A family: Counting past 1, 2 and 3. Immunol. Cell Biol..

[39] Zhang Z., Louboutin J.P., Weiner D.J., Goldberg J.B., Wilson J.M. (2005). Human airway epithelial cells sense *Pseudomonas aeruginosa* infection via recognition of flagellin by Toll-like receptor 5. Infect. Immun..

[40] Pfalzgraff A., Weindl G. (2019). Intracellular Lipopolysaccharide Sensing as a Potential Therapeutic Target for Sepsis. Trends Pharmacol. Sci..

[41] Schleimer R.P. (2017). Immunopathogenesis of Chronic Rhinosinusitis and Nasal Polyposis. Annu. Rev. Pathol..

[42] Hamilos D.L. (2015). Drivers of chronic rhinosinusitis: Inflammation versus infection. J. Allergy Clin. Immunol..

[43] Carey R.M., Freund J.R., Hariri B.M., Adappa N.D., Palmer J.N., Lee R.J. (2020). Polarization of protease-activated receptor 2 (PAR-2) signaling is altered during airway epithelial remodeling and deciliation. J. Biol. Chem..

[44] Carey R.M., Lee R.J. (2019). Taste Receptors in Upper Airway Innate Immunity. Nutrients.

[45] Levit A., Nowak S., Peters M., Wiener A., Meyerhof W., Behrens M., Niv M.Y. (2014). The bitter pill: Clinical drugs that activate the human bitter taste receptor TAS2R14. FASEB J..

[46] Karaman R., Nowak S., Di Pizio A., Kitaneh H., Abu-Jaish A., Meyerhof W., Niv M.Y., Behrens M. (2016). Probing the Binding Pocket of the Broadly Tuned Human Bitter Taste Receptor TAS2R14 by Chemical Modification of Cognate Agonists. Chem. Biol. Drug Des..

[47] Hariri B.M., McMahon D.B., Chen B., Freund J.R., Mansfield C.J., Doghramji L.J., Adappa N.D., Palmer J.N., Kennedy D.W., Reed D.R. (2017). Flavones modulate respiratory epithelial innate immunity: Anti-inflammatory effects and activation of the T2R14 receptor. J. Biol. Chem..

[48] Lee R.J., Xiong G., Kofonow J.M., Chen B., Lysenko A., Jiang P., Abraham V., Doghramji L., Adappa N.D., Palmer J.N. (2012). T2R38 taste receptor polymorphisms underlie susceptibility to upper respiratory infection. J. Clin. Investig..

[49] Roland W.S., Gouka R.J., Gruppen H., Driesse M., van Buren L., Smit G., Vincken J.P. (2014). 6-methoxyflavanones as bitter taste receptor blockers for hTAS2R39. PLoS ONE.

[50] Jaggupilli A., Singh N., De Jesus V.C., Gounni M.S., Dhanaraj P., Chelikani P. (2019). Chemosensory bitter taste receptors (T2Rs) are activated by multiple antibiotics. FASEB J..

[51] Moreau-Marquis S., Redelman C.V., Stanton B.A., Anderson G.G. (2010). Co-culture models of Pseudomonas aeruginosa biofilms grown on live human airway cells. J. Vis. Exp..

[52] Gentzsch M., Mall M.A. (2018). Ion Channel Modulators in Cystic Fibrosis. Chest.

[53] Medapati M.R., Singh N., Bhagirath A.Y., Duan K., Triggs-Raine B., Batista E.L., Chelikani P. (2021). Bitter taste receptor T2R14 detects quorum sensing molecules from cariogenic *Streptococcus mutans* and mediates innate immune responses in gingival epithelial cells. FASEB J..

[54] Jaggupilli A., Singh N., Upadhyaya J., Sikarwar A.S., Arakawa M., Dakshinamurti S., Bhullar R.P., Duan K., Chelikani P. (2017). Analysis of the expression of human bitter taste receptors in extraoral tissues. Mol. Cell. Biochem..

[55] Roland W.S., Vincken J.P., Gouka R.J., van Buren L., Gruppen H., Smit G. (2011). Soy isoflavones and other isoflavonoids activate the human bitter taste receptors hTAS2R14 and hTAS2R39. J. Agric. Food Chem..

[56] Roland W.S., van Buren L., Gruppen H., Driesse M., Gouka R.J., Smit G., Vincken J.P. (2013). Bitter taste receptor activation by flavonoids and isoflavonoids: Modeled structural requirements for activation of hTAS2R14 and hTAS2R39. J. Agric. Food Chem..

[57] Kuroda Y., Ikeda R., Yamazaki T., Ito K., Uda K., Wakabayashi K., Watanabe T. (2016). Activation of human bitter taste receptors by polymethoxylated flavonoids. Biosci. Biotechnol. Biochem..

[58] Behrens M., Gu M., Fan S., Huang C., Meyerhof W. (2017). Bitter substances from plants used in traditional Chinese medicine exert biased activation of human bitter taste receptors. Chem. Biol. Drug Des..

[59] Gocmen J.S., Buyukkocak U., Caglayan O. (2009). In vitro antibacterial activity of some systemic and topical antihistaminic preparations. Clin. Investig. Med..

[60] Areej S., Sattar A., Javeed A., Raza S. (2021). Diphenhydramine and levofloxacin combination therapy against antimicrobial resistance in respiratory tract infections. Future Microbiol..

[61] Hariri B.M., McMahon D.B., Chen B., Adappa N.D., Palmer J.N., Kennedy D.W., Lee R.J. (2017). Plant flavones enhance antimicrobial activity of respiratory epithelial cell secretions against *Pseudomonas aeruginosa*. PLoS ONE.

[62] Simons F.E., Simons K.J. (2011). Histamine and H1-antihistamines: Celebrating a century of progress. J. Allergy Clin. Immunol..

[63] Lee R.J., Chen B., Doghramji L., Adappa N.D., Palmer J.N., Kennedy D.W., Cohen N.A. (2013). Vasoactive intestinal peptide regulates sinonasal mucociliary clearance and synergizes with histamine in stimulating sinonasal fluid secretion. FASEB J..

[64] Howland W.C., Amar N.J., Wheeler W., Sacks H. (2011). Efficacy and safety of azelastine 0.15% nasal spray administered once daily in patients with allergy to Texas mountain cedar pollen. Int. Forum Allergy Rhinol..

[65] Jiao J., Meng N., Zhang L. (2014). The effect of topical corticosteroids, topical antihistamines, and preservatives on human ciliary beat frequency. ORL.

[66] Achterrath-Tuckermann U., Saano V., Minker E., Stroman F., Arny I., Joki S., Nuutinen J., Szelenyi I. (1992). Influence of azelastine and some selected drugs on mucociliary clearance. Lung.

[67] Merkus F.W., Schusler-van Hees M.T. (1992). Influence of levocabastine suspension on ciliary beat frequency and mucociliary clearance. Allergy.

[68] Jiao J., Zhang L. (2019). Influence of Intranasal Drugs on Human Nasal Mucociliary Clearance and Ciliary Beat Frequency. Allergy Asthma Immunol. Res..

[69] Hofmann T., Wolf G., Koidl B. (1998). Effect of topical corticosteroids and topical antihistaminics on ciliary epithelium of human nasal mucosa in vitro. HNO.

[70] Alberty J., Stoll W. (1998). The effect of antiallergic intranasal formulations on ciliary beat frequency of human nasal epithelium in vitro. Allergy.

[71] Ekstedt S., Kumlien Georen S., Cardell L.O. (2020). Effects of MP-AzeFlu enhanced by activation of bitter taste receptor TAS2R. Allergy Asthma Clin. Immunol..

[72] Bruer G.G., Hagedorn P., Kietzmann M., Tohamy A.F., Filor V., Schultz E., Mielke-Kuschow S., Meissner J. (2019). Histamine H1 receptor antagonists enhance the efficacy of antibacterials against Escherichia coli. BMC Vet. Res..

[73] El-Nakeeb M.A., Abou-Shleib H.M., Khalil A.M., Omar H.G., El-Halfawy O.M. (2011). In vitro antibacterial activity of some antihistaminics belonging to different groups against multi-drug resistant clinical isolates. Braz. J. Microbiol..

[74] Mfuna Endam L., Filali-Mouhim A., Boisvert P., Boulet L.P., Bosse Y., Desrosiers M. (2014). Genetic variations in taste receptors are associated with chronic rhinosinusitis: A replication study. Int. Forum Allergy Rhinol..

[75] Kuhn C., Bufe B., Batram C., Meyerhof W. (2010). Oligomerization of TAS2R bitter taste receptors. Chem. Senses.

[76] Kuhn C., Meyerhof W. (2013). Oligomerization of sweet and bitter taste receptors. Methods Cell Biol..

[77] Kim D., Pauer S.H., Yong H.M., An S.S., Liggett S.B. (2016). beta2-Adrenergic Receptors Chaperone Trapped Bitter Taste Receptor 14 to the Cell Surface as a Heterodimer and Exert Unidirectional Desensitization of Taste Receptor Function. J. Biol. Chem..

[78] Gurevich V.V., Gurevich E.V. (2008). How and why do GPCRs dimerize?. Trends Pharmacol. Sci..

[79] Terrillon S., Bouvier M. (2004). Roles of G-protein-coupled receptor dimerization. EMBO Rep..

[80] Freund J.R., Mansfield C.J., Doghramji L.J., Adappa N.D., Palmer J.N., Kennedy D.W., Reed D.R., Jiang P., Lee R.J. (2018). Activation of airway epithelial bitter taste receptors by *Pseudomonas aeruginosa* quinolones modulates calcium, cyclic-AMP, and nitric oxide signaling. J. Biol. Chem..

[81] Wright J.M., Merlo C.A., Reynolds J.B., Zeitlin P.L., Garcia J.G., Guggino W.B., Boyle M.P. (2006). Respiratory epithelial gene expression in patients with mild and severe cystic fibrosis lung disease. Am. J. Respir. Cell Mol. Biol..

[82] Ke Y., Reddel R.R., Gerwin B.I., Miyashita M., McMenamin M., Lechner J.F., Harris C.C. (1988). Human bronchial epithelial cells with integrated SV40 virus T antigen genes retain the ability to undergo squamous differentiation. Differentiation.

[83] Shaul P.W. (2002). Regulation of endothelial nitric oxide synthase: Location, location, location. Annu. Rev. Physiol..

[84] Forstermann U., Sessa W.C. (2012). Nitric oxide synthases: Regulation and function. Eur. Heart J..

[85] Gopallawa I., Freund J.R., Lee R.J. (2021). Bitter taste receptors stimulate phagocytosis in human macrophages through calcium, nitric oxide, and cyclic-GMP signaling. Cell. Mol. Life Sci..

