# Supplementary material for: Cilia Stimulatory and Antibacterial Activities of T2R Bitter Taste Receptor Agonist Diphenhydramine: Insights into Repurposing Bitter Drugs for Nasal Infections
Source: Pharmaceuticals (Basel). 2022 Apr 6;15(4):452. doi: 10.3390/ph15040452 (PMC9025516; doi:10.3390/ph15040452)
Supplement: Supplementary file 1 [file pharmaceuticals-15-00452-s001.zip › pharmaceuticals-1630034-supplementary.pdf]

Supplementary Materials: Kuek, McMahon, *et al*. Immunomodulatory effects of diphenhydramine.

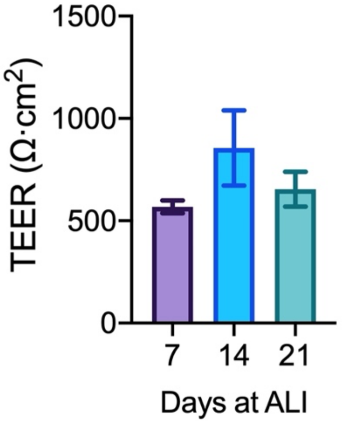

Figure S1: Transepithelial electrical resistance (TEER) of primary air-liquid interface (ALI) nasal cultures. Primary ALI cultures reached a transepithelial electrical resistance (TEER) of ≥500 Ω•cm^2 after 7 days of air exposure which continued over the 3-week period of study. High TEER signifies a tight epithelial monolayer. This, along with appearance of motile cilia (as extensively shown and described in (1)) was used to verify differentiation. Results shown are mean ± SEM from ALIs from 4 individual patients from one group of differentiations. Results are representative of those throughout the study. Background filter resistance was subtracted out using blank transwell filters.

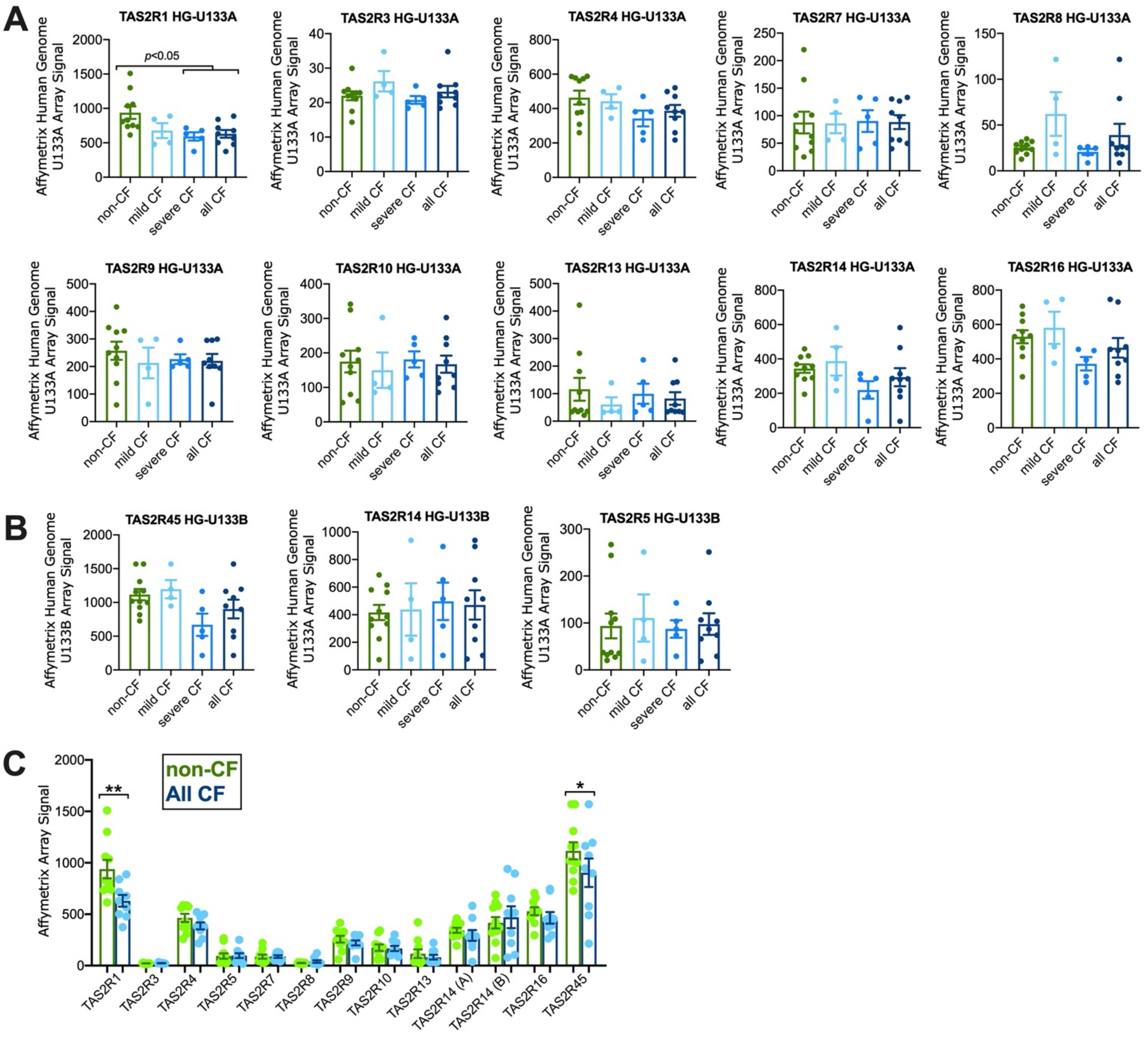

Figure S2: TAS2R gene expression in non-CF vs CF nasal epithelium. Previously analyzed Affymetrix Human Genome U133A and U133B array expression data from (2) was downloaded from NCBI Gene Expression Omnibus (Reference Series GSE2395, data set records GDS2142 and GDS2143). These data analyzed nasal epithelium of non-cystic fibrosis (CF) vs cystic fibrosis (CF) patients with mild or severe lung disease as defined by the investigators. CF patients were homozygous F508del CFTR. **(A-B)** Changes in expression of TAS2R genes encoding individual T2R receptors that were contained in these arrays (HG-U133A array shown in *A* and HG-U133B array shown in *B*) are plotted and analyzed by one-way ANOVA with Dunnett's posttest comparing all values to non-CF. No significant differences were observed except for a decrease in TAS2R1 encoding T2R1 in severe CF and all CF groups. The all CF group is the pool of mild + severe CF groups. Notably, no change in T2R14 was observed on either HG-

U133A or HG-U133B array. **(C)** The relative expression of the TAS2Rs was compared between all CF vs non-CF patients by one way ANOVA with Bonferonni posttest. *TAS2R1* (encoding T2R1) and TAS2R45 (encoding T2R45) were the highest expressed TAS2Rs in this data set and were slightly but significantly decreased in CF patients when compared in this manner. No other T2Rs were significantly different, including T2R14 (shown in two groups as it was included in both arrays. All together, these data support conclusions in the main text that there are minor, if any, differences in TAS2R expression between CF and non-CF nasal epithelial cells.

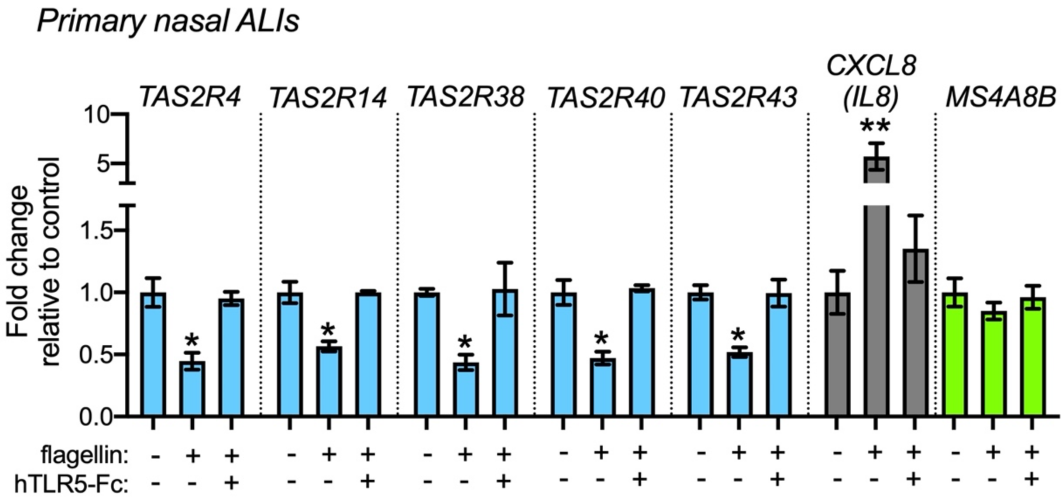

Figure S3: Inhibition of Flagellin effects with hTLR5-Fc. Primary nasal ALIs were stimulated with flagellin (1 μg/mL) as in main text Figure 3A. Expression of TAS2R4, TAS2R14, TAS2R38, TAS2R40, and TAS2R43 genes were decreased. This decrease was reduced in the presence of a TLR5 inhibitor (1 μg/mL recombinant soluble ectodomain of human TLR5, hTLR5-Fc, InvivoGen, San Diego, CA). IL-8 (*CXCL8* gene) was increased, as expected, and this was also reduced with hTLR5-Fc. *MS4A8B* was unaffected by flagellin, showing the effects of flagellin are not a signal of global downregulation of all cilia-associated genes. Significance by one-way ANOVA with Bonferonni posttest comparing all genes to respective control (no flagellin or hTLR5-Fc); **p*<0.05, ***p*<0.01.

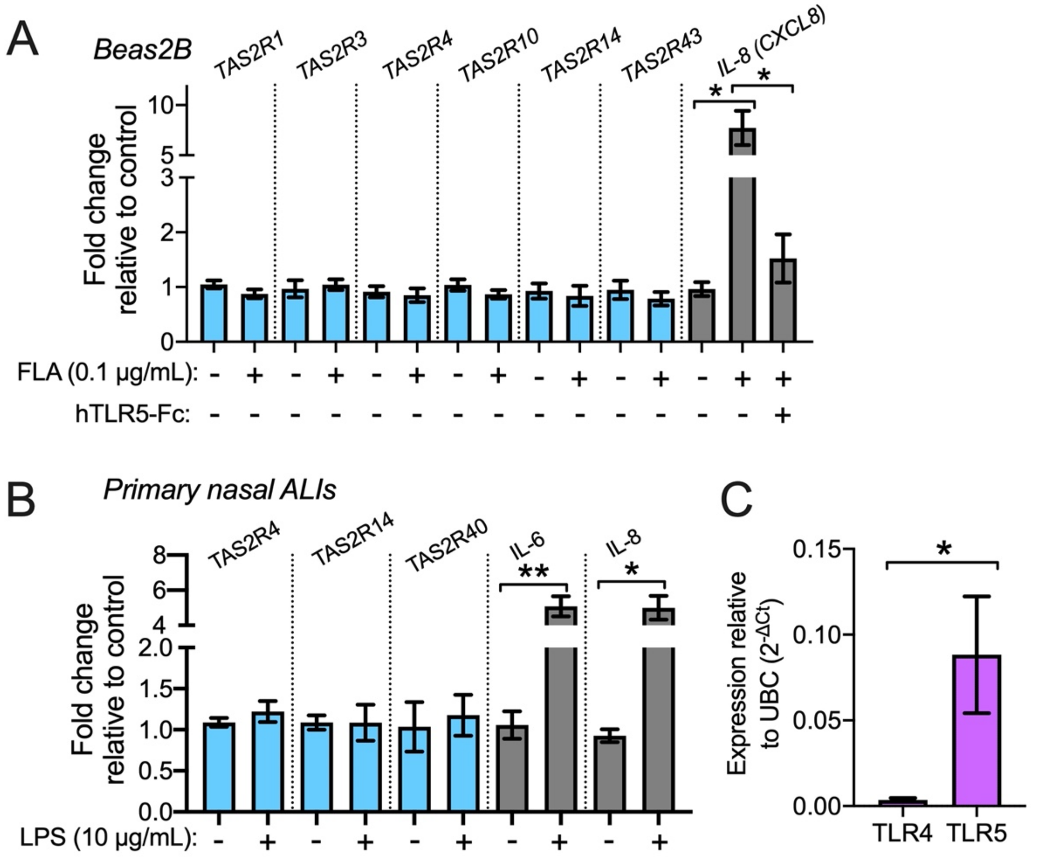

Figure S4: Flagellin-induced changes in TAS2R expression are specific to primary cells and specific to flagellin. (A) Beas-2B cells (adenovirus 12/SV40-immortalized bronchial cells (3)) were treated with flagellin similarly to primary cells in main Figure 1D. No changes in *TAS2R* gene expression were noted among cilia T2R receptors. However, IL-8 increased, as expected, suggesting cells were responsive to TLR5/flagellin signaling. **(B)** Primary nasal ALIs were stimulated with lipopolysaccharide (LPS) instead of flagellin. TAS2R expression did not change but IL-6 and IL-8 increased. Bar graphs in A and B show mean ± SEM of 4 independent experiments (4 individual patients in B). Significance determined by one-way ANOVA with Bonferroni posttest; *p<0.05 and **p<0.01. **(C)** Differentiated primary nasal ALIs, in the absence of any inflammatory stimuli, expressed significantly more TLR5 mRNA compared with TLR4 mRNA. Significance by Student's *t* test; *p<0.05.

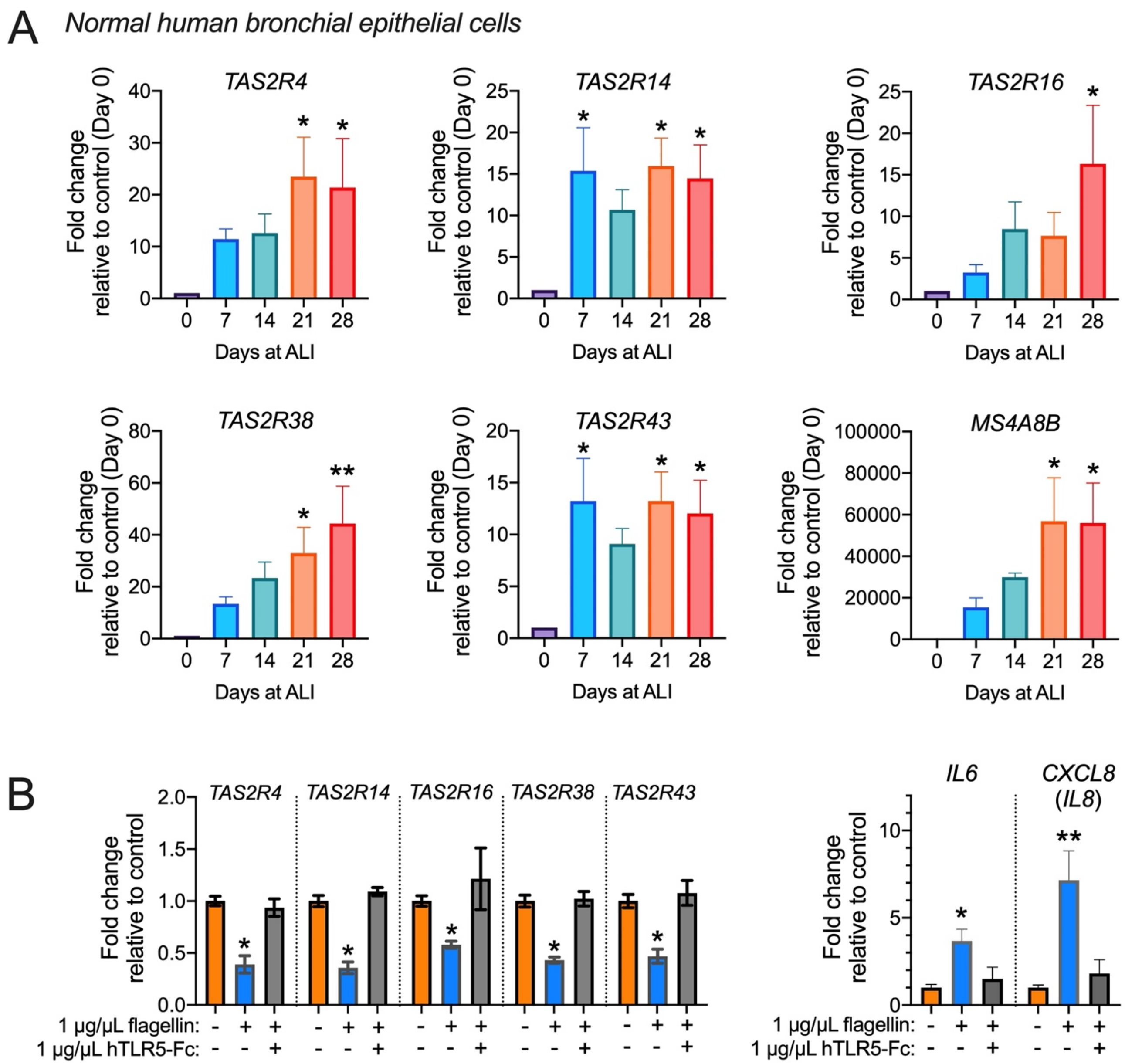

Figure S5: ***TAS2R* gene expression in bronchial cells.** Primary bronchial air-liquid interface cultures (ALIs) were grown from commercially available primary normal human bronchial epithelial (HBE) cells (Lonza, Walkerville MD) exactly as described for nasal cells in the main text. Culture conditions (media, seeding density, etc.) were identical. **(A)** T2R expression increases during differentiation. Ciliary T2Rs, 4, 14, 16, 38, and 43 are encoded by *TAS2R4*, *TAS2R14*, *TAS2R16*, *TAS2R38*, and *TAS2R43* genes. The mRNA expression increased with at least 3 weeks of differentiation as revealed by qPCR. Data points represent the average of 5 patients; significance was analyzed by one-way ANOVA using Dunnett's posttest (* p<0.05). Data points represent the average of 5 patients; significance was analyzed by one-way ANOVA using Dunnett's posttest (* p<0.05). **(B)** TLR5 agonist *Pseudomonas* flagellin was applied to 3

week differentiated primary bronchial ALIs as described in Fig 1 of the main text for primary nasal ALIs. Flagellin. Cultures were treated with various concentrations of flagellin (0.1, 1, or 10 μg/mL) for 24 hours prior to RNA extraction and cDNA analysis via qPCR. Flagellin reduced expression of TAS2R4, TAS2R14, TAS2R16, TAS2R38, and TAS2R43 genes. Co-application of a TLR5 inhibitor (recombinant soluble ectodomain of human TLR5, hTLR5-Fc, InvivoGen, San Diego, CA) blocked this effect. Flagellin increased expression of NFκB-driven *IL6* and *CXCL8* genes encoding IL-6 and IL-8, respectively.

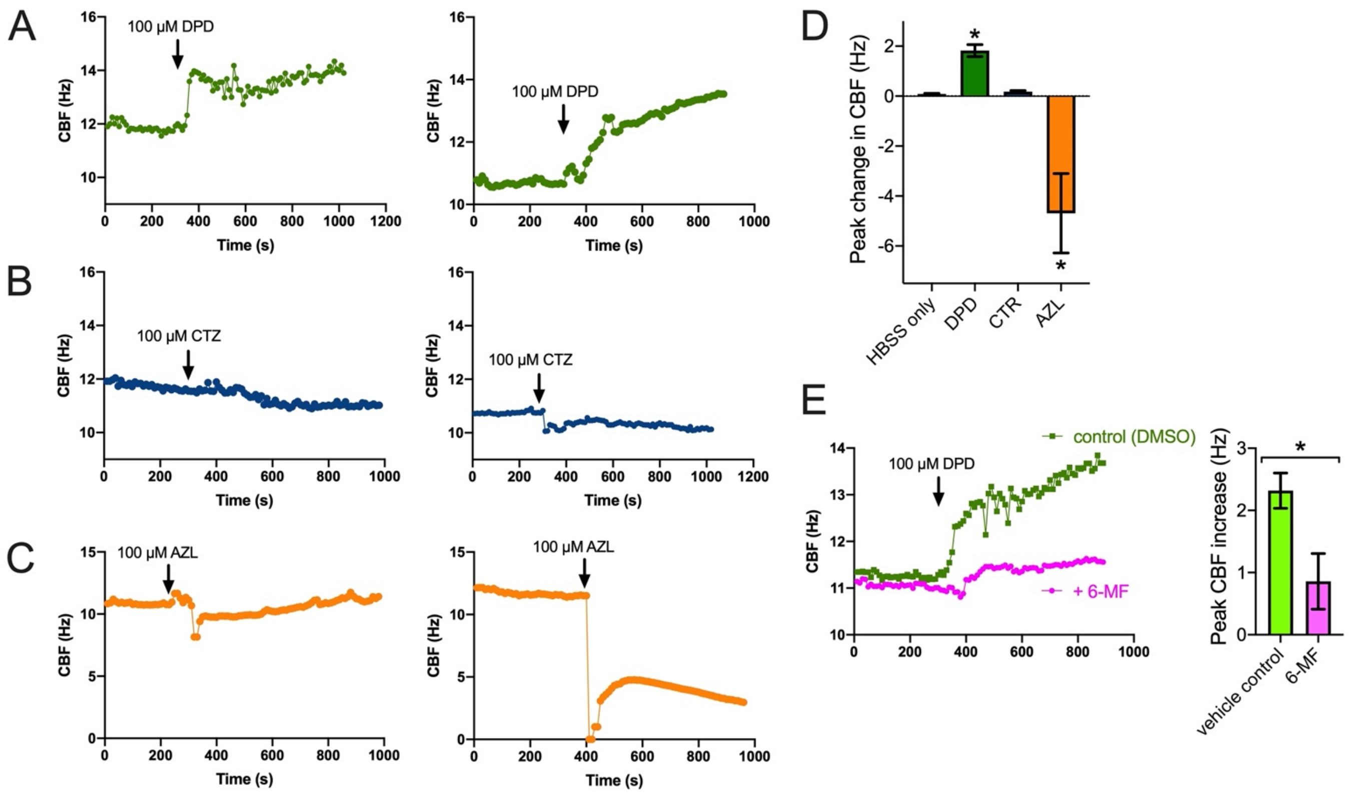

Figure S6: Diphenhydramine (DPD) increases ciliary beat frequency while cetirizine (CTZ) and azelastine (AZL) do not. (A-C) Representative ciliary beat frequency (CBF) traces from ALIs from two individual patients showing responses to 100 μM DPD (A), CTZ (B), or AZL (AZL). **(D)** Bar graph showing peak CBF change in response to indicated drugs or HBSS buffer only control. Significance by one way ANOVA with Dunnett's posttest; *p<0.05. **(E)** Traces and bar graph showing CBF changes in response to 100 μM DPD after treatment with 6-methoxyflavanone (500 μM, treatment as in (4)) or vehicle control (0.1% DMSO).

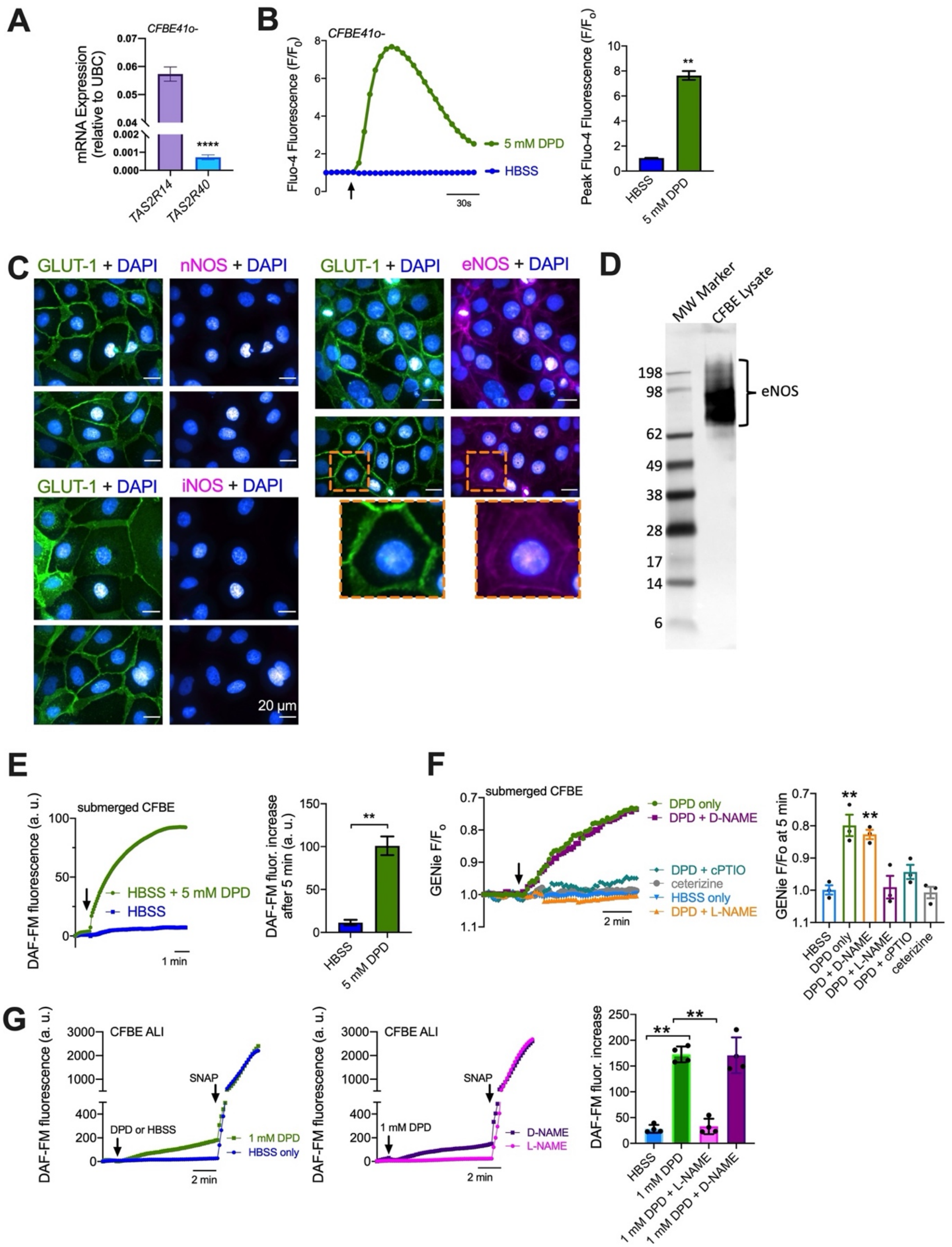

Figure S7: Diphenhydramine (DPD) responses in CFBE41o- (CFBE) cells. (A) qPCR revealed higher expression of *TAS2R14* (encoding T2R14) compared with *TAS2R40* (encoding T2R40) in CFBE cells, fitting with observations of primary nasal (this study) and bronchial cells (5). **(B)** 5 mM DPD activated

Ca^{2+} elevations (measured by Fluo-4) in submerged CFBE cells. Hank's balanced salt solution (HBSS) only was added as control. (A-B) Bar graph shows mean ± SEM of n = 3 independent experiments analyzed by Student's *t* test; ***p*<0.01, *****p*<0.0001. **(C)** Immunofluorescence images of GLUT-1 (membrane marker) plus co-staining for nNOS, iNOS, or eNOS in submerged CFBEs. Representative images shown are taken at the same microscope settings, suggesting membrane staining for eNOS but not nNOS or iNOS. Note that eNOS is palmitoylated and often localized either to the plasma membrane and/or Golgi (6, 7). We previously observed Golgi localization of eNOS in A549 cells with this antibody (8). Plasma membrane localization in CFBE cells may have implications for eNOS activation that are beyond the scope of this current study. These data do, however, support expression of eNOS in CFBEs. **(D)** Western blot analysis revealed eNOS expression in CFBE cells. Western blotting was carried out as described (5). Briefly, cultured CFBE cells were scrapped in PBS then re-suspended in RIPA buffer followed by lysis via sonicating water bath for 4 rounds of 30s pulses. After centrifugation at 800 x *g* for 8 min at 4°C, the supernatant was collected, and protein concentration estimated via BioRad DC Protein Assay (Hercules, CA). Molecular weight marker SeeBlue Plus2 (Thermo Fisher) and 60 µg of cell extract was separated across a 4-12% NuPAGE Bis-Tris gel (Invitrogen) then transferred to a nitrocellulose membrane for subsequent western blot analysis using an eNOS/NOS Type III primary antibody (Fisher Scientific, Cat #610296) and an anti-mouse secondary antibody (Cell Signaling Cat #7076). Together with IF, Western data also supports expression of eNOS in CFBE cells. **(E)** Submerged CFBE cells exhibited increases in DAF-FM fluorescence (likely reflecting NO production) in response to 5 mM DPD. Traces are representative experiments and bar graph shows mean ± SEM of 4 independent experiments analyzed by Student's t test; ***p*<0.01, HBSS only added as control. **(F)** Submerged CFBE cells were transduced with BacMam encoding Green GENie cGMP biosensor (Montana Molecular, Bozeman, MT), previously used to measure cGMP in airway cells (5) and macrophages (9). This biosensor gets dimmer as cGMP increases. We observed a decrease in F/F_0 reflective of an increase in cGMP (plotted inversely so it the trace tracks cGMP). The increase in cGMP was inhibited by NO scavenger cPTIO (Cayman Chemical) or NOS inhibitor L-NAME (10 µM; 30 min pre-treatment) but not inactive D-NAME. Bar graphs shows change in F/F_0 after 5 min stimulation. Significance determined by one-way ANOVA with Dunnett's posttest comparing all values to control (HBSS alone). **(G)** CFBE cells grown at ALI exhibited increases in DAF-FM fluorescence in response to 1 mM DPD that were inhibited by NOS inhibitor L-NAME (10 µM; 30 min pre-treatment) but not inactive analogue D-NAME (10 µM; 30 min pre-treatment). Traces are representative experiments and bar graph shows mean ± SEM of 4 independent experiments analyzed by one way ANOVA with Bonferroni posttest; ***p*<0.01. S-Nitroso-N-Acetyl-D,L-Penicillamine (SNAP; 20 µM; Cayman Chemical) was added at the end of each experiment as a non-specific S-nitrosothiol NO donor to validate that the loaded DAF-FM can detect NO production, further supporting that the DAF-FM changes reflect NO production.

Supplementary References

1. Carey RM, Freund JR, Hariri BM, Adappa ND, Palmer JN, Lee RJ. Polarization of protease-activated receptor 2 (PAR-2) signaling is altered during airway epithelial remodeling and deciliation. J Biol Chem. 2020;295(19):6721-40.

2. Wright JM, Merlo CA, Reynolds JB, Zeitlin PL, Garcia JG, Guggino WB, et al. Respiratory epithelial gene expression in patients with mild and severe cystic fibrosis lung disease. Am J Respir Cell Mol Biol. 2006;35(3):327-36.

3. Ke Y, Reddel RR, Gerwin BI, Miyashita M, McMenamin M, Lechner JF, et al. Human bronchial epithelial cells with integrated SV40 virus T antigen genes retain the ability to undergo squamous differentiation. Differentiation. 1988;38(1):60-6.

4. Jaggupilli A, Singh N, De Jesus VC, Gounni MS, Dhanaraj P, Chelikani P. Chemosensory bitter taste receptors (T2Rs) are activated by multiple antibiotics. FASEB J. 2019;33(1):501-17.

5. McMahon DB, Kuek LE, Johnson ME, Johnson PO, Horn RLJ, Carey RM, et al. The bitter end: T2R bitter receptor agonists elevate nuclear calcium and induce apoptosis in non-ciliated airway epithelial cells. Cell Calcium. 2022;101:102499.

6. Shaul PW. Regulation of endothelial nitric oxide synthase: location, location, location. Annu Rev Physiol. 2002;64:749-74.

7. Forstermann U, Sessa WC. Nitric oxide synthases: regulation and function. Eur Heart J. 2012;33(7):829-37, 37a-37d.

8. Hariri BM, McMahon DB, Chen B, Freund JR, Mansfield CJ, Doghramji LJ, et al. Flavones modulate respiratory epithelial innate immunity: anti-inflammatory effects and activation of the T2R14 receptor. J Biol Chem. 2017;292(20):8484-97.

9. Gopallawa I, Freund JR, Lee RJ. Bitter taste receptors stimulate phagocytosis in human macrophages through calcium, nitric oxide, and cyclic-GMP signaling. Cell Mol Life Sci. 2021;78(1):271-86.
